# Human Cytomegalovirus Nuclear Egress Proteins Ectopically Expressed in the Heterologous Environment of Plant Cells are Strictly Targeted to the Nuclear Envelope

**DOI:** 10.3390/v8030073

**Published:** 2016-03-10

**Authors:** Christian E. Lamm, Katrin Link, Sabrina Wagner, Jens Milbradt, Manfred Marschall, Uwe Sonnewald

**Affiliations:** 1 Division of Biochemistry, Department of Biology, Friedrich-Alexander University Erlangen-Nuremberg, Staudtstrasse 5, Erlangen 91058, Germany; christian.lamm@fau.de (C.L.); katrin.link@web.de (K.L.); 2 Institute for Clinical and Molecular Virology, Friedrich-Alexander University Erlangen-Nuremberg, Schloßgarten 4, Erlangen 91054, Germany; sabrina.wagner@viro.med.uni-erlangen.de (S.W.); jens.milbradt@viro.med.uni-erlangen.de (J.M.); manfred.marschall@viro.med.uni-erlangen.de (M.M.)

**Keywords:** human cytomegalovirus, pUL50, pUL53, nuclear envelope, plant cells, re-initiation supporting protein RISP

## Abstract

In all eukaryotic cells, the nucleus forms a prominent cellular compartment containing the cell’s nuclear genome. Although structurally similar, animal and plant nuclei differ substantially in details of their architecture. One example is the nuclear lamina, a layer of tightly interconnected filament proteins (lamins) underlying the nuclear envelope of metazoans. So far no orthologous lamin genes could be detected in plant genomes and putative lamin-like proteins are only poorly described in plants. To probe for potentially conserved features of metazoan and plant nuclear envelopes, we ectopically expressed the core nuclear egress proteins of human cytomegalovirus pUL50 and pUL53 in plant cells. pUL50 localizes to the inner envelope of metazoan nuclei and recruits the nuclear localized pUL53 to it, forming heterodimers. Upon expression in plant cells, a very similar localization pattern of both proteins could be determined. Notably, pUL50 is specifically targeted to the plant nuclear envelope in a rim-like fashion, a location to which coexpressed pUL53 becomes strictly corecruited from its initial nucleoplasmic distribution. Using pUL50 as bait in a yeast two-hybrid screening, the cytoplasmic re-initiation supporting protein RISP could be identified. Interaction of pUL50 and RISP could be confirmed by coexpression and coimmunoprecipitation in mammalian cells and by confocal laser scanning microscopy in plant cells, demonstrating partial pUL50-RISP colocalization in areas of the nuclear rim and other intracellular compartments. Thus, our study provides strong evidence for conserved structural features of plant and metazoan nuclear envelops and identifies RISP as a potential pUL50-interacting plant protein.

## 1. Introduction

Although already observed in the beginning of the 18th century by the Dutch microscopist Antonie van Leeuwenhoek, the nature of the eukaryotic nucleus still raises numerous questions especially in plants and is subject to extensive studies. Superficially, animal and plant nuclei are similar but they differ in their compositional details. This can be seen already in the constitution of the nuclear envelope (NE): In both kingdoms, the structure consists of a double membrane layer that delimits the cytoplasm and the nucleoplasm. The outermost of both nuclear membranes is continuous with the endoplasmic reticulum (ER). In both kingdoms the nuclear pore complexes (NPCs) regulate a multifaceted mutual exchange of metabolites and macromolecules across the nuclear envelope [1]. However, NPCs are not identical structures in plant and animal cells. Some NPC proteins are conserved at the amino acid sequence but others are only functionally and structurally related [2,3,4].

These evolutionary differences in nuclear architecture are not restricted to the NPC, but are also found in the protein composition of the nuclear envelope and its associated compartments, with one of the best examples being the nuclear lamina. This structure directly underlying the inner nuclear membrane (INM) literally is a meshwork of proteins, mainly comprised of lamins and various types of lamin-associated proteins [5,6,7]. The manifold functions of this lattice include supporting and shaping of the nucleus [8,9,10], as well as tethering of heterochromatin to the nuclear periphery [6,11,12]. Other functional aspects range from DNA damage repair and replication to cellular signal transduction [13,14,15]. Interestingly, on the basis of primary sequence similarity, no plant lamin genes have been identified so far. However, the existence of a mesh-like structure similar to the nuclear lamina of metazoan cells has been demonstrated by electron microscopy in tobacco nuclei [16], and a number of nuclear proteins were identified and discussed as proteins with similar secondary structure, localization and molecular functions, but varying amino acid sequence as compared to metazoan lamins. In order to emphasize both similarities and differences, the postulated plant equivalent to the metazoan lamina has been referred to as *plamina* [17,18,19,20,21].

In this context, the transmembrane Sad1/UNC-84 proteins (SUN) are important to mention since these proteins are among the few NE constituents sharing an evolutionary conserved domain and can be found likewise in metazoans, plants and fungi [22]. In the animal kingdom, SUN domain-containing proteins were found to localize at the inner nuclear membrane in trimeric arrangements and to interact with lamins via their amino termini. [23,24,25,26,27]. The use of Förster resonance energy transfer microscopy (FRET) illustrated that plant SUN proteins target the NE and could be shown to associate with a plant lamin-like protein [28,29]. In metazoa, SUN proteins are able to bind outer nuclear membrane proteins, designated KASH (Klarsicht, ANC-1 and SYNE homology proteins), thereby building a bridge across the nuclear envelope, which enables interactions with actin filaments, microtubules, intermediate filaments or motor proteins [24,30,31]. Of note, no known KASH homologs have been identified in plants based on primary sequence comparisons, but functional analogs were postulated adopting some related functionality of KASH-like proteins. *Arabidopsis thaliana* WPP domain-interacting proteins (WIPs) could be shown to interact with both *Arabidopsis thaliana* SUN1 and SUN2, an interaction which is necessary to retain WIP effectively at the NE [32]. Unlike metazoan KASH proteins, these WIPs are not directly linked to the cytoskeleton, but seem to be dependent on the interaction with other plant outer nuclear membrane proteins. Previous analyses identified a WIP interaction with WPP domain-interacting tail-anchored proteins (WITs), which themselves are able to bind myosin XI-I, further stabilizing the association with the cytoskeleton [33].

These examples illustrate that the nuclear envelope of plant and animal cells might not be directly conserved on the level of primary amino acid sequences, but that functional homologs may exist that express functional redundancy conferred by evolutionary divergent complexes.

To address the question whether functional similarities exist between metazoan and plant nuclear envelops, we employed a heterologous protein expression approach, in which human viral proteins were used as baits to identify candidate proteins associated with the nuclear envelope and to investigate the conservation of nuclear molecular functions. Human cytomegalovirus (HCMV) is a double-stranded DNA virus of the family *Herpesviridae*, which replicates in the host cell nucleus. This nuclear phase includes the formation of viral nucleocapsids, the packaging with viral genomic DNA and a subsequent nucleocytoplasmic transition of particles to overcome the nuclear envelope. Several steps of subsequent extranuclear, cytoplasmic trafficking are following to finally result in the cellular release of infectious progeny virions. However, prior to the budding through both inner and outer nuclear membranes, viral particles have to reorganize the proteinaceous portions of the nuclear envelope [34,35]. With the nuclear pores being far too narrow to allow for direct export of the 130 nm capsids, HCMV has evolved a strategy of regulated nuclear capsid egress [36,37]. To achieve this task, HCMV assembles a protein complex consisting of both viral and cellular components at the nuclear envelope, the so called nuclear egress complex (NEC). In mammalian cells, the multimeric NEC is able to mediate lamin phosphorylation, which in turn leads to destabilization and disassembly of the nuclear lamina. It has been suggested that the cellular peptidyl-prolyl cis/trans isomerase Pin1 induces a conformational change of nuclear lamins that promotes the disassembly process of the nuclear lamina in HCMV-infected cells [38]. The core NEC is formed by the two conserved viral proteins pUL50 and pUL53, which heterodimerize and remain anchored to the inner nuclear membrane by a carboxy-terminal transmembrane domain of pUL50 [39,40,41]. Together, these viral effectors are thought to provide a basis for further recruitment of protein factors necessary for the destabilization of the lamina, such as protein kinases, bridging factors and functionally uncharacterized components. Among these are the cellular kinase PKCα, the viral kinase pUL97, the inner nuclear membrane protein emerin and the multifunctional cellular protein p32/gC1qR [42,43,44,45,46,47,48,49]. In this study, we provide first evidence that a molecular mechanism enabling INM-retention is conserved across kingdoms, and that the nucleoplasmic localization of pUL53 as well as its pUL50-mediated nuclear rim recruitment is surprisingly similar in plant and human cells. Furthermore, we identified a pUL50-interacting plant protein, re-initiation supporting protein (RISP, locus identifier AT5G61200), which upon coexpression with pUL50 associates to granular structures at the plant nuclear envelope. The conclusions drawn from the observed patterns of protein interaction and translocation in plant cells as well as their possible significance for understanding nuclear plant architecture are discussed.

## 2. Materials and Methods

### 2.1. Plant Materials

*Nicotiana benthamiana* plants were grown in soil in a greenhouse maintaining a 16 h light/8 h darkness photoperiod and temperatures of 25 and 20 °C, respectively.

### 2.2. Plasmid Constructs

For expression in plants—the coding sequences of pUL50 and pUL53 were amplified by PCR from pcDNA-UL50-HA and pcDNA-UL53-FLAG [43], respectively, using specific oligonucleotide primers (sequences given in Table 1). In both cases the Gateway^®^ Cloning system (Thermo Fisher Scientific, Waltham, MA, USA) was used: After subcloning of the PCR products into the vector pENTR/D-TOPO^®^, the final constructs were generated using the LR-Clonase^®^‎ Enzyme Mix and the destination vectors pK7WGF2 [50] and pGWB660 [51] for pUL50 and pUL53 respectively, leading to eGFP::pUL50 and pUL53::tagRFP fusion proteins.

To generate a RFP-destination vector, the sequence of tagRFP was amplified from pGWB660 [51] using appropriate oligopeptide primers (sequences given in Table 1). The resulting amplicon was subcloned into the entry vector pCR™-Blunt (Thermo Fisher Scientific), excised by the restriction enzymes SalI and PstI and inserted into the vector pRB35S [52], giving rise to the vector 35S-X:RFP. The RISP coding sequence was amplified from *Arabidopsis thaliana* cDNA and cloned in the same manner (restriction enzymes BamHI and SalI) and inserted into the latter vector, resulting in a RISP::tagRFP construct.

The expression of all constructs was driven by the constitutively active cauliflower mosaic virus (CaMV) 35S promoter, whereas termination of transcription was achieved by the CaMV 35S terminator, the nopaline synthase terminator and the octopine synthase terminator for eGFP::pUL50, pUL53::tagRFP and RISP::tagRFP, respectively.

For expression in yeast—a plasmid coding for pUL50 in fusion with the GAL4 DNA-binding domain (GAL4BD-UL50) was described previously [43].

For expression in human cells—a plasmid coding for RISP with a C-terminal FLAG-tag was generated by PCR amplification of the RISP open reading frame. Standard PCR amplification was performed using pENTR-RISP as template with oligonucleotide primers purchased from Biomers (Ulm, Germany); sequences of oligonucleotides are given in Table 1. After cleavage with EcoRV and NotI, the PCR product was inserted into the mammalian expression vector pcDNA3.1(+) (Thermo Fisher Scientific). The mammalian expression constructs pcDNA-UL50(1-358)-HA, pcDNA-UL53-HA, pcDNA-UL53-FLAG and pcDNA-UL97-HA have been described previously [43,53]. Construct pDsRed1-N1, expressing the red fluorescent protein (RFP), was purchased from BD Biosciences Clontech (Heidelberg, Germany) and was used as a positive control for transfection experiments in mammalian cells.

Expression of both RISP-FLAG and pUL50(1-358)-HA was driven by the HCMV immediate early promoter/enhancer (PCMV), while termination was achieved by the bovine growth hormone polyadenylation signal (BGHpA).

### 2.3. Transient Expression in Nicotiana benthamiana and Confocal Laser Scanning Microscopy

Transient expression was achieved through pressure infiltration of *Agrobacterium tumefaciens* cultures as described. In case of RISP::tagRFP expressions, a tomato bushy stunt virus p19-construct, which was kindly provided by Kathrin Paulus, Friedrich-Alexander University Erlangen-Nürnberg, was coinfiltrated to minimize post transcriptional gene silencing [54].

For confocal microscopy, leaf segements of *N. benthamiana* were cut and the abaxial sides were observed. Pictures were obtained using a Leica TCS SP5 II confocal laser scanning microscope, fluorescent dyes and proteins were excited using lasers with wavelengths of 405 nm, 488 nm and 561 nm for DAPI, eGFP and tagRFP, respectively. To prevent fluorescence bleed through during expression of multiple fluorescent proteins, sequential scans were conducted.

Life cell DAPI-stainings were achieved by leaf infiltration of a 1 mg/mL DAPI (4′,6-diamidino-2-phenylindole) solution. After 10 min, leaves were flushed by infiltration of water and subsequently observed under the microscope. When statistical analysis were employed, the LAS AF software (Leica Microsystems, Wetzlar, Germany was used. Scale bars for the figures depicting the regions of interest used for colocalization in the supplementary materials were introduced with FIJI [55].

### 2.4. Transient Expression in Human Epithelial Hela Cells, Indirect Immunofluorescence Assay and Confocal Laser-Scanning Microscopy

Human epithelial cells (HeLa) were seeded on coverslips for transient transfection experiments. Cells were transfected using Lipofectamine 2000 (Thermo Fisher Scientific) with eukaryotic expression plasmids coding for hemagglutinin (HA)-tagged pUL50 (pcDNA-UL50-HA) and FLAG-tagged pUL53 (pcDNA-UL53-FLAG) according to the manufacturer’s protocols. At 2 days post-transfection, cells were fixed and permeabilized followed by indirect immunofluorescence staining. Stainings were performed as described previously [43,48] using primary antibodies rabbit polyclonal antibody (pAb), anti-HA (HA.11; Eurogentec Deutschland GmbH, Cologne, Germany) and Alexa Fluor 488-/555-conjugated secondary antibodies (Molecular Probes, Eugene, OR, USA). Cells were mounted using DAPI-containing Vectashield mounting medium (Vector Laboratories, Burlingame, CA, USA). Images were acquired by sequential scanning using a TCS SP5 confocal laser-scanning microscope (Leica Microsystems) and analyzed using LAS AF software (Leica Microsystems).

### 2.5. Plant Protein PAGE and Western Blot Analysis

To obtain a total protein extract, two leaf discs (Diameter appr. 9 mm) were frozen in liquid nitrogen, ground in 70 µL extraction buffer containing urea (225 mM Tris HCl pH6.8, 50% glycerol, 5% SDS, 250 mM DTT, 8 M urea, 0.02% bromophenol blue) and centrifuged for 3 min at 15,900× *g*. Supernatant was loaded on a Bis/Tris gel containing 12% acrylamide for electrophoresis. Proteins were transferred onto a nitrocellulose membrane, which afterwards was blocked for at least 1 h in blocking buffer (20 mM Tris HCl, 500 mM NaCl, 0.1% (*v*/*v*) Tween 20, 5% (*w*/*v*) milk powder). Detection was carried out using suitable primary antibodies and horseradish peroxidase-conjugated secondary antibodies, followed by enhanced chemiluminescence detection. Nitrocellulose membrane stainings were conducted using a solution of 0.5% (*w*/*v*) Ponceau S in 1% acetic acid. Membranes were incubated for 2 min in the staining solution and subsequently destained in distilled water.

### 2.6. Yeast Two-Hybrid Screening

For yeast two-hybrid screening, the GAL4-based [56] Clontech Matchmaker 3 system (Clontech, Heidelberg, Germany) was used. In short, the vector pGBT9 containing a GAL4 DNA-binding domain in frame with pUL50 was chosen to transform the yeast strain Y187 [57] using the previously described PEG/Lithium acetate/ssDNA method [58]. As a cDNA library, an *Arabidopsis thaliana* library in AH109^b^ yeast cells obtained from tair [59] was used. After mating both strains and plating on minimal medium, yeast colonies were obtained after 5–7 days. X-Gal filter staining assays were conducted to verify positive interactions [60]. The identity of positive clones was revealed by plasmid preparation and following sequencing.

### 2.7. Coimmunoprecipitation Assay

Human embryonic kidney epithelial cells (293T) were cultivated and cotransfected with expression plasmids coding for HA- or FLAG-tagged cytomegaloviral proteins with FLAG-tagged RISP by the use of polyethlyeniminine-DNA complexes as described previously [48,61]. Three days post-transfection, cells were used for protein-protein interaction experiments utilizing coimmunoprecipitation (CoIP). Immunoprecipitation was performed under previously described conditions using 2 µL of mouse monoclonal antibody (mAb) anti-HA (12CA5; Roche Applied Science, Risch, Switzerland) [43]. CoIP samples and expression controls (input) taken prior to the addition of the CoIP antibody were subjected to standard Western blot analysis using mouse mAb anti-FLAG (M2; Sigma, St. Louis, MO, USA) and rabbit polyclonal antiserum (pAb) anti-HA (HA.11; Covance Inc., Princeton, NJ, USA).

## 3. Results

### 3.1. HCMV pUL50 Localizes to the Plant Nuclear Envelope

**Figure 1:**
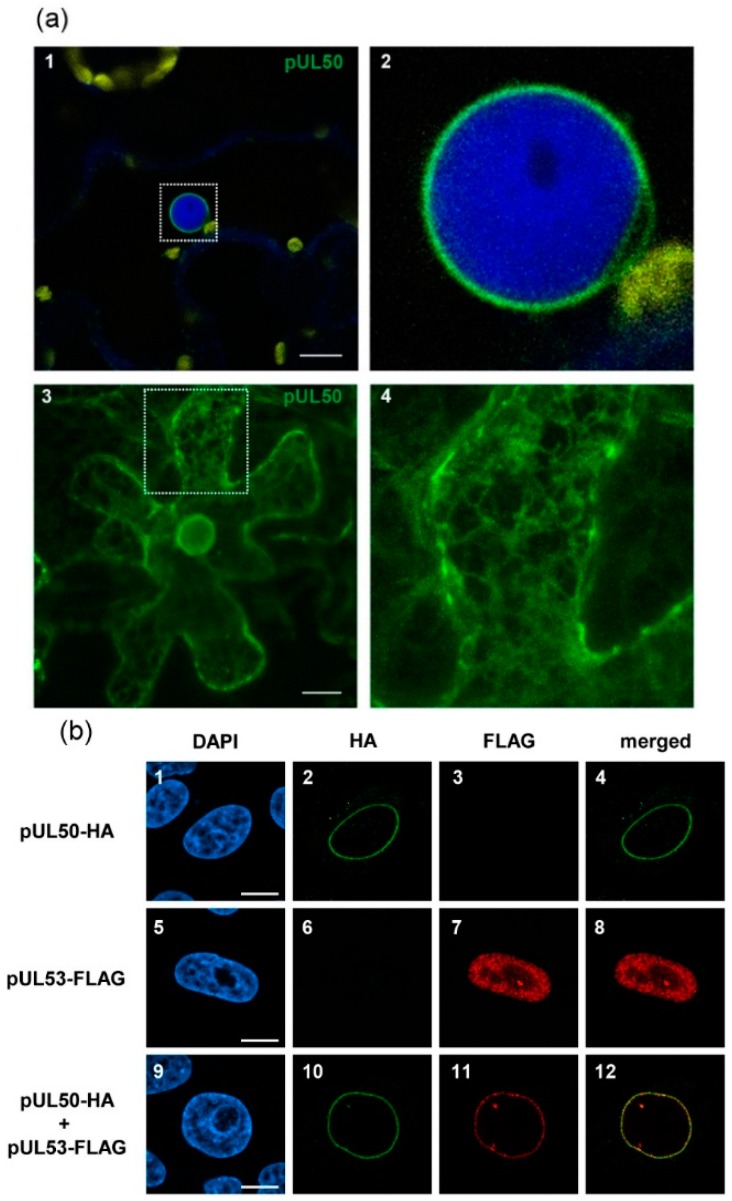
Expression and localization of pUL50 (**a**) Transient expression of eGFP::pUL50 in *Nicotiana benthamiana*: panel 1, DAPI-staining was performed to identify nuclei (blue), the GFP-fusion construct (green) was localized to the nuclear envelope; chloroplast autofluorescence is shown in yellow, the dotted box indicates the section shown in panel 2; panel 2, magnified section from panel 1; panel 3, pUL50 also localized to the endoplasmic reticulum in *Nicotiana benthamiana*, maximum projection of a z-stack image, the dotted box indicates the section shown in panel 4; panel 4, magnified section from panel 3 reveals the localization of eGFP::pUL50 to the net-like structure of the endoplasmic reticulum; (**b**) Immunolocalization of pUL50-HA (green) and pUL53-FLAG (red) in HeLa cells: Panels 1-4, single transfection of pUL50-HA in DAPI (blue) stained cells revealed the localization to the nuclear envelope; panels 5-8, single transfection of pUL53-FLAG in DAPI stained cells showed nucleoplasmic localization; panels 9-12, cotransfection of pUL50-HA and pUL53-FLAG demonstrating the potential of pUL50 to recruit pUL53 to the nuclear rim. White bars represent 10 µm.

In order to investigate the architecture of plant nuclei, the herpesviral protein pUL50 was chosen for heterologous expression experiments, as the protein is localized to the inner nuclear membrane in human cells [43,48]. For localization studies, eGFP was fused to the N-terminus of pUL50 (Figure S1a). The construct was transiently expressed in leaves of *Nicotiana benthamiana* using Agrobacterium tumefaciens-mediated transformation. The integrity of eGFP::pUL50 could be confirmed by Western blot analysis. Using mouse monoclonal antibody (mAb) directed against pUL50 (mAb-UL50; kindly provided by Stipan Jonjic and Tihana Lenac, University of Rijeka, Rijeka, Croatia), a band at the corresponding size of *ca*. 70 kDa was detected, verifying proper protein expression (Figure S1b). When observed under a confocal laser-scanning microscope two days after Agrobacterium infiltration, eGFP::pUL50 showed a clear and continuous localization to the nuclear envelope, indistinguishable from the physiological rim localization of the native protein (Figure 1b, panels 1–4). A DAPI staining served as a marker confirming the localization at the nuclear periphery in plant cells (Figure 1a, panels 1–2). Noteworthy, eGFP::pUL50 also localized to the endoplasmic reticulum, which can be seen in a maximum projection of a z-stack image series (Figure 1a, panels 3,4). Interestingly, an initial localization of pUL50 to the endoplasmic reticulum was also described in HCMV-infected human cells, which is, during the course of viral replication, abolished by pUL53-mediated INM-retention of pUL50 [62].

### 3.2. pUL50 is Able to Recruit HCMV pUL53 to the Nuclear Periphery in Nicotiana benthamiana

**Figure 2:**
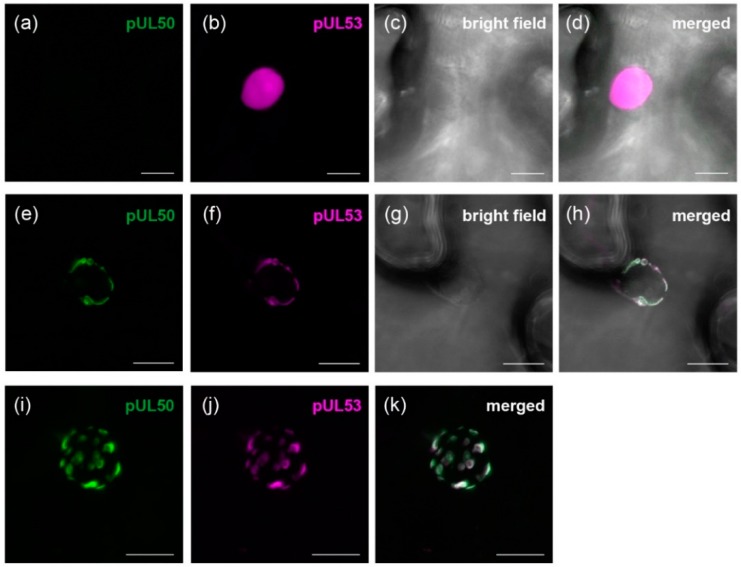
Expression of pUL53::tagRFP (depicted in magenta) and colocalization with eGFP::pUL50 (depicted in green). (**a**)–(**d**) Transient expression of pUL53::tagRFP in *Nicotiana benthamiana*: No fluorescence in the GFP-channel, tagRFP-signal corresponding to pUL53 was restricted to the nucleoplasm; (**e**)–(**h**) Coexpression of eGFP::pUL50 and pUL53::tagRFP in *Nicotiana benthamiana*: Colocalization of both constructs suggested that pUL50 was able to recruit pUL53 to the nuclear rim; (**i**)–(**k**) Coexpression of eGFP::pUL50 and pUL53::tagRFP in *Nicotiana benthamiana*, z-stack maximum projection: Constructs colocalized in patch-like structures at the nuclear periphery. White bars represent 10 µm.

In order to investigate pUL50-pUL53 interaction in coexpressing plant cells, a fusion protein for pUL53 was generated carrying a C-terminally tagged RFP (Figure S2a). Autofluorescent pUL53::tagRFP was transiently expressed in *Nicotiana benthamiana* leaves to determine its localization. The correct expression of the construct was verified by Western blot analysis, showing a band at the corresponding size of appr. 70 kDa (Figure S2b). Confocal laser scanning microscopy revealed that pUL53::tagRFP localized at various intranuclear sites also including the nucleolus (Figure 2a–d). For comparison with the human cellular localization, pUL50 and pUL53 were transfected in HeLa cells in parallel. During single transfection, pUL53 was targeted to the nucleus (Figure 1b, panels 5–8) but, in contrast to plant cells, pUL53 did not exhibit a nucleolar accumulation and was also not as dispersed as in *Nicotiana benthamiana*. Upon coexpression, both fluorescent proteins strictly colocalized in *Nicotiana benthamiana*. Importantly, eGFP::pUL50 was able to recruit pUL53::tagRFP from its even nuclear localization to the nuclear rim (Figure 2e–h). This is in accordance with the situation in HeLa cells, where a cotransfection of both viral constructs led to a very similar result (Figure 1b, panels 9–12). Noteworthy, in Nicotiana benthamiana, both signals were not evenly distributed across the nuclear envelope, but localized to a patch-like pattern at the nuclear rim. This pattern was particularly distinct when depicted through the maximum projection of a z-stack image series (Figure 2i–k).

### 3.3. pUL50 Interacts with Arabidopsis thaliana re-Initiation Supporting Protein (RISP)

**Figure 3:**
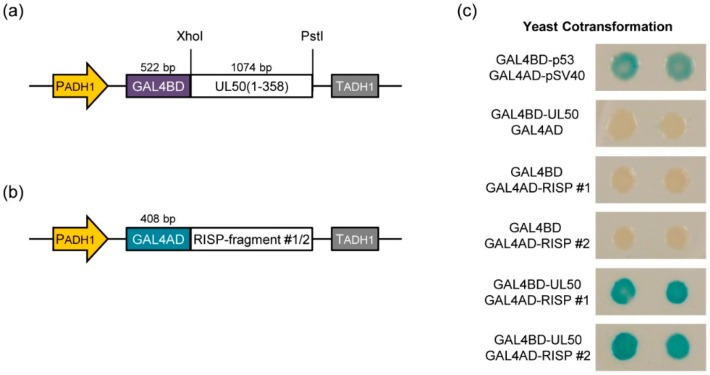
Yeast two-hybrid assay with pUL50 as bait. (**a**) Bait construct yielding a GAL4 binding domain-pUL50 fusion protein, used for screening and cotransformation. PADH, alcohol dehydrogenase 1 promoter; TADH1, alcohol dehydrogenase 1 terminator; restriction sites were used as indicated; values over schemes indicate size of the respective fragment in basepairs; (**b**) Prey construct identified during screening and used for cotransformation, the GAL4 activation domain fused to a fragment of *Arabidopsis thaliana* RISP; (**c**) X-Gal filter staining assay confirms interaction of pUL50 and RISP in yeast cells.

Given the nuclear envelope localization of eGFP::pUL50 in *Nicotiana benthamiana*, candidate plant proteins possibly interacting with pUL50 were investigated. To this end, a yeast two-hybrid screening was performed using an *Arabidopsis thaliana* cDNA library fused to the GAL4 activation domain, employing a truncated version of pUL50 (amino acids 1–358, lacking its C-terminal transmembrane domain) fused to the GAL4 DNA-binding domain as a bait protein (Figure 3a). After mating of the yeast clones either transformed with the cDNA library or the bait construct, the cells were plated on selection medium to determine the efficiency of the mating process. In total, approximately 3.4 × 10^6^ mating events were observed. Yeast cells expressing their reporter genes (HIS3 and lacZ) and thus containing potentially interacting constructs were selected by the use of minimal medium and X-Gal filter staining assays. Hereby, we identified six individual yeast clones, which were further analyzed by plasmid preparation and sequencing. All six clones harbored the identical prey plasmid, containing a fragment of the coding sequence of *Arabidopsis thaliana* re-initiation supporting protein (RISP, locus identifier AT5G61200). In previous works, the plant-specific RISP was described to interact with a cauliflower mosaic virus protein, transactivator viroplasmin (TAV), in order to support the translation re-initiation of polycistronic mRNA. As a postulated part of the translation machinery, RISP was found to be present in dot-like aggregates in the cytoplasm [63]. To verify the result from the yeast two-hybrid screen, two of the six rescued plasmids (Figure 3b) were cotransformed with pGAL4BD::pUL50 in yeast cells and X-Gal filter staining assays were performed. As negative controls, the respective empty vectors were used, whereas the SV40 large T antigen in pGAD424 and p53 in pGBT9 were cotransformed as positive control (both supplied by the manufacturer). While the cotransformation with the negative controls did not lead to staining of the yeast colonies upon X-Gal treatment, the interaction of SV40 T antigen and p53 resulted in an expression of the reporter genes and thus to a blue staining of the colonies. In case of pUL50 and RISP, all colonies of the tested duplicates exhibited a strong blue staining, indicating a solid interaction of both proteins in yeast, confirming the previous result (Figure 3c).

**Figure 4:**
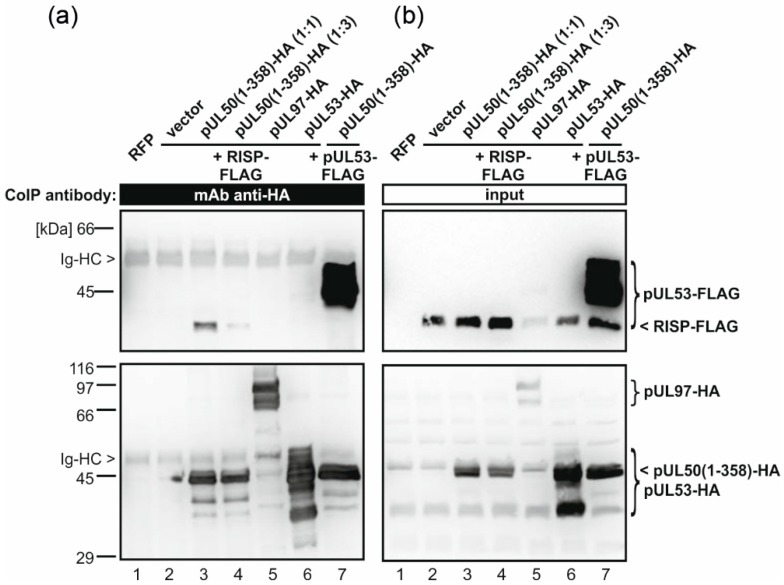
Interaction between RISP with HCMV pUL50 in human cells. (**a**,**b**) Coimmunoprecipitation (CoIP) assay. Human 293T cells were transiently cotransfected with constructs coding for FLAG-tagged RISP and HA-tagged HCMV proteins pUL50(1–358) (ratio 1:1 and 1:3, lanes 3 and 4, respectively), pUL97 (lane 5) or pUL53 (lane 6), or with an empty vector (pcDNA3.1, lane 2) as control. Coexpression of HA-tagged pUL50(1–358) and FLAG-tagged pUL53 served as CoIP control (lane 7); RFP expression served as transfection control (lane 1). At 2 days post-transfection, cells were lysed, and HA-tagged proteins were immunoprecipitated using mouse monoclonal antibody mAb-HA. CoIP samples (**a**) and lysate controls (input) (**b**) taken prior to the addition of the CoIP antibody were subjected to standard Western blot analysis using tag-specific antibodies as indicated. Ig-HC, cross-reactive band for immunoglobulin heavy chain.

To verify the results obtained in the yeast two-hybrid system, we analyzed the putative interaction between RISP and HCMV pUL50 by coexpression and coimmunoprecipitation (CoIP) in human embryonic kidney 293T cells. Therefore, we first generated a construct encoding FLAG-tagged RISP for expression in mammalian cells (Figure S3). Mammalian expression constructs for a truncated version of pUL50 (*i.e.*, amino acids 1–358; lacking its C-terminal transmembrane domain) (Figure S3) and further HCMV control proteins were generated previously [43]. The HCMV proteins pUL50(1–358), pUL53 or pUL97 were coexpressed with RISP in 293T cells. For protein-protein interaction analysis, hemagglutinin (HA)-tagged viral proteins were immunoprecipitated with a tag-specific antibody. CoIP samples and lysate controls were analyzed by Western blotting (Figure 4a,b). Importantly, RISP was specifically coimmunoprecipitated along with pUL50 (Figure 4a, lanes 3–4), whereas CoIP of RISP by other HCMV control proteins was negative (Figure 4a, lanes 5–6). Together, these results confirmed the interaction of RISP with HCMV pUL50.

### 3.4. pUL50 Colocalizes with Arabidopsis thaliana Re-Initiation Supporting Protein (RISP)

To investigate wether the subcellular localization of RISP is in agreement with the interaction with the herpesviral protein when expressed in plants, we generated a fusion protein consisting of RISP and tagRFP (Figure S4). Subsequently, the RISP::tagRFP fusion protein was transiently coexpressed with eGFP::pUL50 in *Nicotiana benthamiana*. To overcome post transcriptional gene silencing of the RISP construct, the tomato bushy stunt virus silencing suppressor p19 was also coexpressed [54]. After two days, the respective fluorescence signals were monitored by confocal laser scanning microscopy.

**Figure 5:**
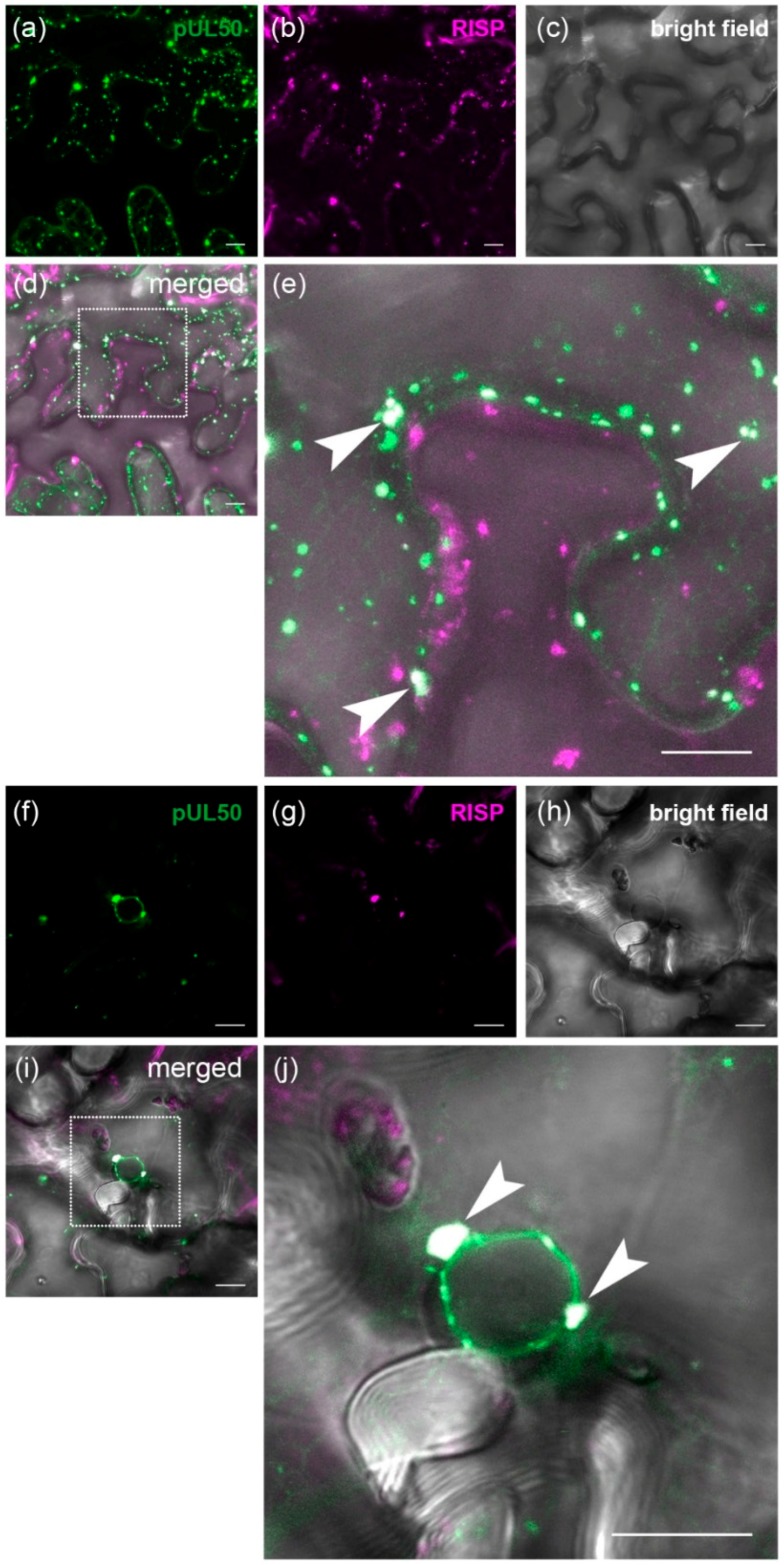
Colocalization studies of eGFP::pUL50 (depicted in green) and RISP::tagRFP (depicted in magenta). (**a**) Note that upon coexpression, eGFP::pUL50 was found retained in cytoplasmic aggregates; (**b**) As reported earlier [63], RISP::tagRFP was mainly localized to cytoplasmic dot-like structures; (**d**) The merged image demonstrates colocalization of eGFP::pUL50 and RISP::tagRFP. The dotted box indicates the section magnified in (**e**); (**e**) Magnified section from (**d**), arrowheads point towards some of the cytoplasmic aggregates that incorporate both eGFP- and tagRFP-fluorescence signals; (**f**) During coexpression with RISP::tagRFP, eGFP::pUL50 still localized to the nuclear rim, in part also in nucleus-associated aggregates; (**g**) RISP::tagRFP localized to aggregates in the vicinity of the nucleus; (**i**) Both eGFP::pUL50 and RISP::tagRFP signals overlapped to a substantial extent at nuclear sites. The dotted box represents the area magnified in (**j**), in which the colocalization becomes obvious, arrowheads indicate aggregates of eGFP- and tagRFP-signals that merge in association with the nuclear envelope; white bars represent 10 µm.

In accordance with observations of Thiébeauld *et al.* [63], RISP::tagRFP was found to localize in cytoplasmic aggregates (Figure 5b). Confirming the preceding results from the yeast two-hybrid screen as well as the coimmunoprecipitation, eGFP::pUL50 could be found in cytoplasmic dot-like structures (Figure 5a), of which most were colocalizing with the RISP-aggregates (Figure 5d,e). We used Pearson’s correlation coefficient as a statistical tool for quantifying colocalization. Therefore, a region of interest (ROI) was defined enclosing the whole cell of the inset shown in Figure 5e (see Figure S5), since this cell coexpressed both eGFP::pUL50 and RISP::tagRFP. A Pearson’s correlation coefficient of 0.53 at a colocalization rate of 60.4% demonstrated a strong colocalization between pUL50 and RISP according to the classification by Zinchuk *et al.* [64].

Apart from these aggregates, eGFP::pUL50 still decorated the nuclear rim in coexpressing cells (Figure 5f), while RISP::tagRFP was also present in dots of varying size (Figure 5g). Similar to the cytoplasmic aggregates, we observed a colocalization of eGFP::pUL50 and RISP::tagRFP also at the nuclear rim (Figure 5i,j). Here again, a Pearson’s correlation coefficient of 0.85 at a colocalization rate of 89.1% for a ROI enclosing the nucleus (Figure S6) pointed to a very strong colocalization. Taken together, our data demonstrate an interaction of pUL50 with RISP and that this interaction is not only restricted to the cytoplasm, but is also present at the nuclear envelope, which is the actual site of action of pUL50.

## 4. Discussion

### 4.1. A Mechanism Similar to Herpesviral Nuclear Retention of pUL50 is Conserved in Plants

**Figure 6:**
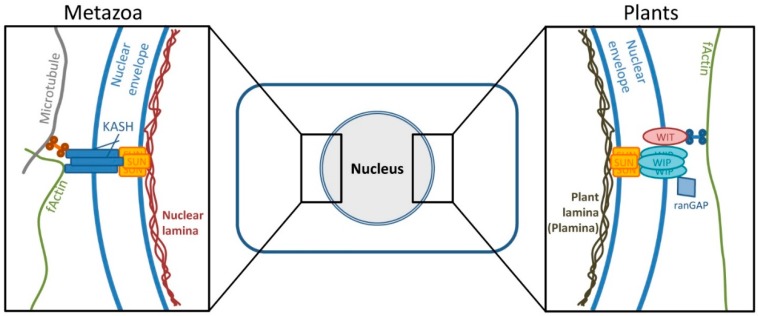
Comparison of selected structures from nuclear envelopes of metazoans and plants. Although not conserved on the level of amino acid sequence, several structures share functional redundancy. Depicted is the protein complex linking the nucleo- and cytoskeleton as well as the nuclear lamina/plant lamina (plamina). fActin: Filamentous actin; KASH: Klarsicht, ANC-1 and SYNE homology proteins; SUN: Sad1/UNC-84 proteins; WIP: WPP domain-interacting proteins; WIT: WPP domain-interacting tail-anchored proteins.

Although superficially similar, the nuclei of animals and plants differ greatly in their structural constitution. For instance, the metazoan intermediate filaments known as lamins are able to polymerize to form a mesh-like structure underneath the inner nuclear membrane, a process which is facilitated by their tripartite domain architecture consisting of an amino-terminal globular domain, a central alpha-helical rod domain and highly conserved carboxy-terminal tail domain [5,65,66]. This lamin layer is the basis for further interactions, one example being the association with the inner nuclear membrane SUN proteins, which themselves are able to interact with the outer nuclear membrane KASH proteins, which may associate with the cytoskeleton. Due to this close functional interconnection between the nuclear lamina and the cytoskeleton, the SUN-KASH complex has also been considered as a linker between the nucleoskeleton and the cytoskeleton (LINC) [24,30,31]. In plants however, solely the SUN proteins are conserved on a primary sequence level, whereas the functions of the other mentioned proteins seem to be adopted by structurally unrelated, but functionally redundant proteins (Figure 6).

In order to gain insights into the architecture and functionality of plant nuclei, we employed a heterologous expression approach. Utilizing two HCMV proteins, pUL50 and pUL53, we aimed to compare the conservation of nuclei across kingdoms and to identify potential candidate proteins being important for plant nuclear processes. In human cells, pUL50 was shown to target the nuclear envelope and to recruit pUL53 from a nucleoplasmic localization towards the nuclear rim to form a multiprotein complex consisting of both viral and cellular proteins [43,48].

Our experiments revealed that pUL50 is, when expressed in leaves of *Nicotiana benthamiana*, able to reach the nucleus and to decorate the NE in a fashion similar to the situation observed in human cells. The fact that the protein was also detected at the endoplasmic reticulum corroborates the hypothesis that pUL50 is able to target a conserved mechanism: Notably, pUL50 does not possess a nuclear localization signal (NLS), but has to rely on another mechanism to reach the nuclear envelope [62]. In particular, pUL50 is present in a speckled pattern at early time points of HCMV infection, which was presumed to represent a pUL50 localization in endosomes or at the endoplasmic reticulum. It is tempting to speculate that an initial ER-localization of pUL50 indicates that the protein is synthesized at the ER, from where it travels alongside the ER-membrane, the outer nuclear membrane and the pore membrane to reach its final destination, the INM [67].

In case of pUL50, we previously suggested that interaction with the NLS-containing pUL53 accounts for nuclear retention of both proteins at later time points of infection [62]. In fact, these observations could be fortified by the present study, as both the ER-localization, as well as retention of pUL50 by pUL53 could be observed using the heterologous expression approach. At the same time, our results are evidence for the kingdom-spanning presence of this inner nuclear membrane retention mechanism for transmembrane proteins, which might also be applicable for plant INM-proteins that do not encode a NLS.

### 4.2. Interaction of pUL50 with the Arabidopsis thaliana protein RISP

Using the yeast two-hybrid system, one potential interaction partner of pUL50 could be identified from an *Arabidopsis thaliana* cDNA library. With 3.4 × 10^6^ yeast mating events, the efficiency of the screening was sufficiently high. The fact that only a single candidate was found can be attributed to one of several reasons: Either the yeast system is not suitable to identify further interactors with nuclear or nuclear membrane localization, or RISP is *de facto* the only protein present in the library to interact with pUL50. It could also be possible that the respective cDNA of a theoretical interactor is not represented in the cDNA library used for the screening. However, the interaction was confirmed by two independent experimental approaches: It was possible to pull down RISP together with pUL50 by coimmunoprecipitation in human cells. In addition, a solid colocalization of both proteins could be detected by confocal laser scanning microscopy in *Nicotiana benthamiana* leaf epidermis cells.

To date, the interacting *Arabidopsis* protein is only poorly characterized. It was first described in 2009, where it could be shown to interact with the cauliflower mosaic virus protein transactivator viroplasmin (TAV) to enhance its function in translation re-initiation of polycistronic mRNA. Furthermore, it interacts with components of the protein translation machinery, namely with the ribosomal 60S subunit and the translation initiation factor eIF3 [63]. The authors also investigated the subcellular localization of RISP using both a fluorescent protein fusion construct as well as immunolabelling in tobacco BY-2 cells and found it to be dispersed throughout the cytoplasm in a granular manner.

In case of the interaction with pUL50 shown in this study, RISP colocalized with the HCMV protein in cytoplasmic aggregates, as expected for this cellular factor. However, our findings uncouple the plant protein from an exclusive function in translation re-initiation, as the used eGFP::pUL50 construct was not designed as a polycistronic construct dependent on re-initiation mechanisms. Instead, a more general role of RISP during protein translation could be imagined, for instance to provide enhanced stability for the translation machinery.

On the other hand, the interaction may not be due to a required translational mechanism, but induced by the herpesviral protein itself. However, as RISP is not conserved across kingdoms, it is challenging to relate the interaction with pUL50 with processes necessary for HCMV infection. In fact, no association of pUL50 with viral translation processes is known.

In addition to a cytoplasmic colocalization, RISP-pUL50 complexes could also be detected in aggregates of varying size in the vicinity of the nuclear envelope. Considering the ectopic nature of the viral protein, this might be a condition that has no contribution to nuclear processes and is merely a consequence of the demonstrated interaction of pUL50 and RISP. Contrariwise, this specific localization might as well hint towards a role of RISP not only in translational processes in the observed cytoplasmic granules, but also in the surrounding of the nuclear membrane system, which is the actual residence of pUL50. Whether the function of RISP in this region is diverging from its postulated role in protein translation remains unclear. In addition, the known mammalian proteins interacting with pUL50 do not provide indications on the possible task of the plant protein during this heterologous expression, as cellular proteins like the protein kinase PKCα, the INM structural protein emerin, p32/gC1qR (adaptor for viral kinase pUL97) or endophilin-A2 (postulated role during NE destabilization) share no obvious functional redundancy with RISP [42,43,44,45,46,47,48,49].

Our present study provides a first molecular insight into viral-cellular protein complexes arising from ectopic heterologous coexpression that are strictly translocated to the plant nuclear envelope. This model system may provide a tool for further compositional and functional investigations of this so far poorly characterized plant cell compartment.

## Figures and Tables

**Table 1:** Sequences of oligonucleotide primers used for cloning.

Primer Name	Sequence (5’ to 3’)
**Primers for RFP Vector Construction**	
RFP fw SalI	GTCGACGGATCTGGTGTGTCTAAGGGCGAAGAGCTG
RFP rev PstI	CTGCAGCTATCAATTAAGTTTGTGCCCCAGTTTG
**Primers for Localization Studies**	
***in Plants***	
pUL50 fw	CACCATGGAGATGAACAAGGTTCTCCATC
pUL50 rev	TCAGTCGCGGTGTGCGGAG
pUL53 fw	CACCATGTCTAGCGTGAGCGGC
pUL53 rev	AGGCGCACGAATGCTGTTGAG
RISP fw	GGATCCAACAATGTCGAGTAATTGGGGAAGTAGCTCG
RISP rev	GTCGACTACAGCAGGAAGAGGAACTAAGCAAGTTG
***in Human Cells***	
5-RISP_EcoRV-Acc65I	TCAGGTACCGATATCATGAACGACCTGAGTGAACATGTAC
3-RISP-FLAG_NotI-ApaI	TCAGGGCCCGCGGCCGCTTACTTGTCGTCATCGTCTTTGTAGTCTACAGCAGGAAGAGGAACTAAGC

## Supplementary Materials

The following are available online at www.mdpi.com/1999-4915/8/3/73/s1, Figure S1: eGFP::pUL50 construct and Western blot detection, Figure S2: pUL53::tagRFP construct and Western blot detection, Figure S3: CoIP constructs, Figure S4: RISP::tagRFP construct, Figure S5,S6: Regions of interest used for statistical analysis.
