# Supplementary material for: Human Cytomegalovirus Nuclear Egress Proteins Ectopically Expressed in the Heterologous Environment of Plant Cells are Strictly Targeted to the Nuclear Envelope
Source: Viruses. 2016 Mar 10;8(3):73. doi: 10.3390/v8030073 (PMC4810263; doi:10.3390/v8030073)
Supplement: Supplementary file 1 [file viruses-08-00073-s001.pdf]

Supplementary Materials: Human Cytomegalovirus Nuclear Egress Proteins Ectopically Expressed in the Heterologous Environment of Plant Cells are Strictly Targeted to the Nuclear Envelope

Christian Lamm, Katrin Link, Sabrina Wagner, Jens Milbradt, Manfred Marschall and Uwe Sonnewald

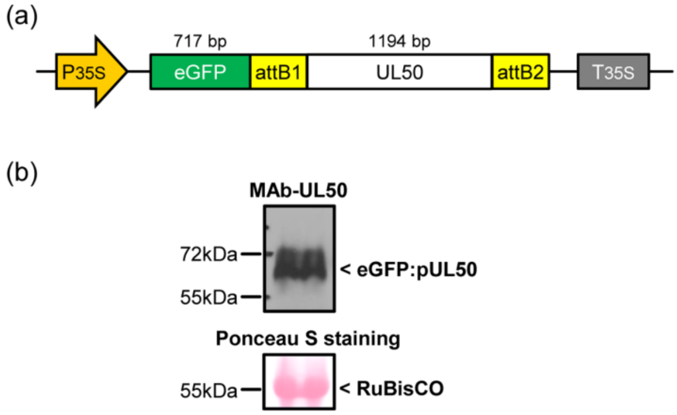

Figure S1. eGFP::pUL50 construct and Western blot detection. (**a**) Schematic representation of the eGFP::pUL50 construct. P35S, CaMV 35S promoter; attB1/2, Gateway® technology recombination sites; T35S, CaMV 35S terminator; values over scheme indicate size of the respective fragment in basepairs; (**b**) eGFP::pUL50 protein expression verified via Western blot detection using a monoclonal antibody directed against pUL50; Ponceau S staining as protein loading control showed a proper band for the highly abundant ribulose-1,5-bisphosphat-carboxylase/-oxygenase (RuBisCO).

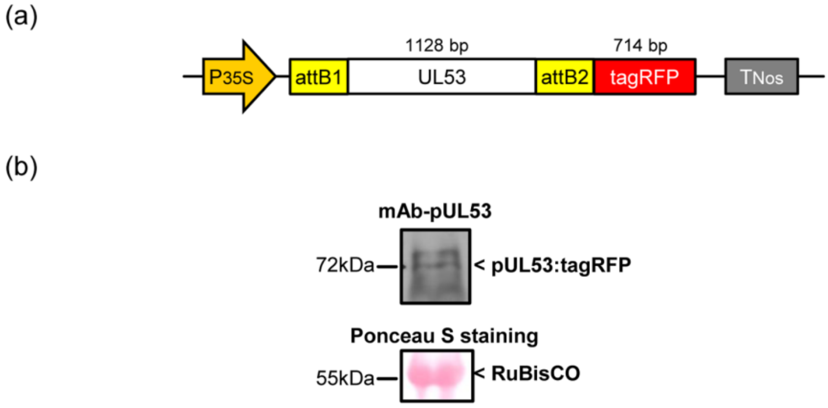

Figure S2. pUL53::tagRFP construct and Western blot detection. (**a**) Schematic representation of the pUL53::tagRFP-construct. P35S, CaMV 35S promoter; attB1/2, Gateway® technology recombination sites; TNos, nopaline synthase terminator; values over schemes indicate size of the respective fragment in basepairs; (**b**) pUL53::tagRFP protein expression verified via Western blot detection using a monoclonal antibody directed against pUL53; Ponceau S staining as protein loading control showed a proper band for the highly abundant ribulose-1,5-bisphosphat-carboxylase/-oxygenase (RuBisCO).

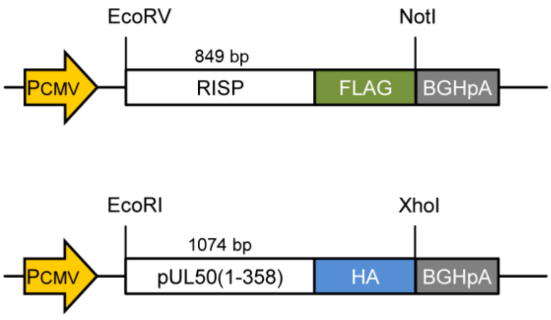

Figure S3. CoIP constructs. Constructs encoding FLAG-tagged RISP and HA-tagged pUL50 (amino acids 1–358) for expression in mammalian cells. PCMV, HCMV immediate early promoter/enhancer; BGHpA, bovine growth hormone polyadenylation signal; restriction sites were used as indicated; values over schemes indicate size of the respective fragment in basepairs.

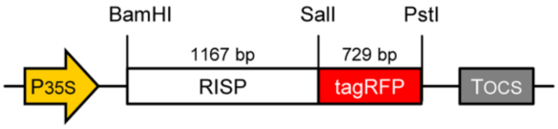

Figure S4. RISP::tagRFP construct. (**a**) Schematic representation of the RISP::tagRFP construct. P35S, CaMV 35S promoter; TOCS, octopine synthase terminator; restriction sites were used as indicated; values over schemes indicate size of the respective fragment in basepairs.

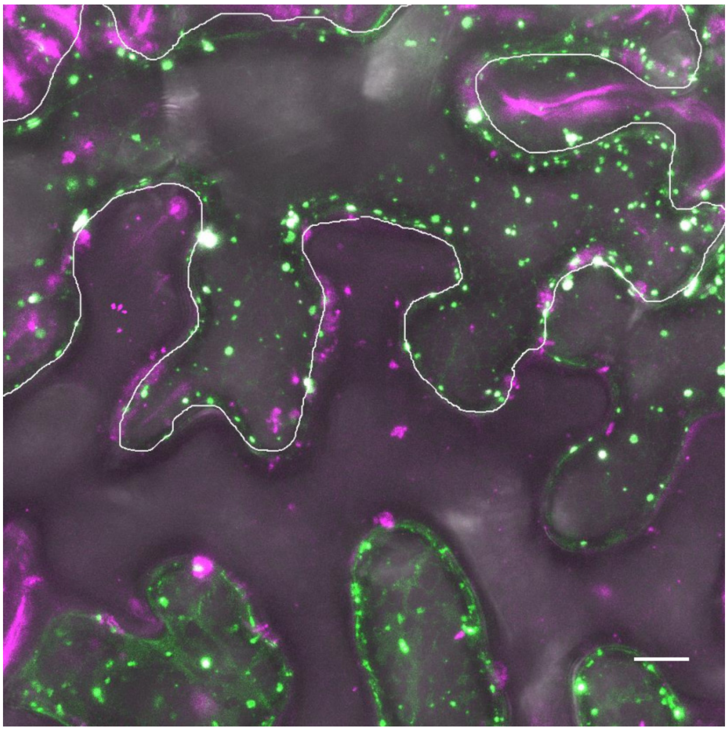

Figure S5. Region of interest used for statistical analysis. A cell coexpressing both RISP::tagRFP and eGFP::pUL50 was selected using the ROI-tool to calculate colocalization rate and Pearson's correlation coefficient using LAS AF software (Leica Microsystems). Scale bar, 10µm.

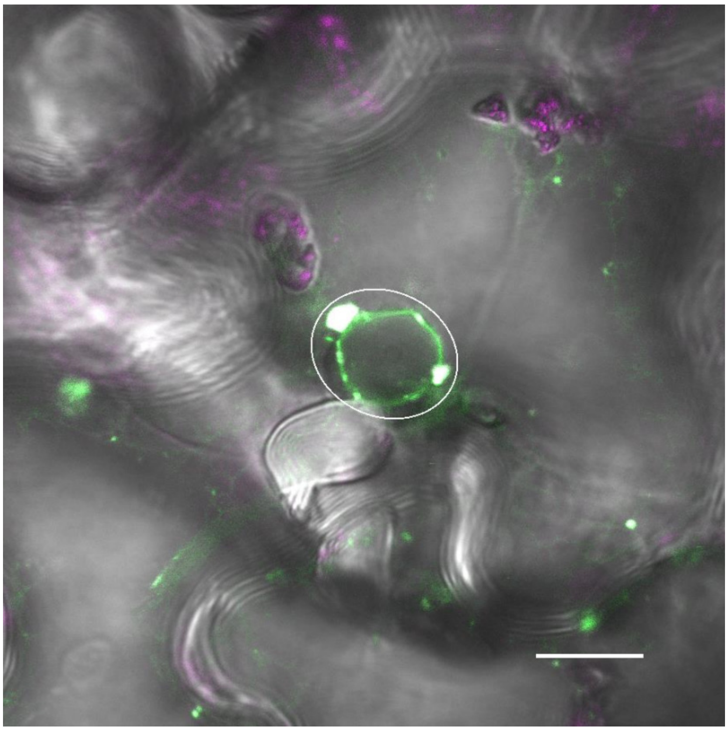

Figure S6. Region of interest used for statistical analysis. A ROI enclosing the nucleus was set, which was used for calculation of colocalization rate and Pearson's correlation coefficient using LAS AF software (Leica Microsystems). Scale bar, 10μm.

© 2016 by the authors; licensee MDPI, Basel, Switzerland. This article is an open access article distributed under the terms and conditions of the Creative Commons by Attribution (CC-BY) license (http://creativecommons.org/licenses/by/4.0/).
